# Supplementary material for: miR-128 inhibits telomerase activity by targeting TERT mRNA
Source: Oncotarget. 2018 Jan 19;9(17):13244–53. doi: 10.18632/oncotarget.24284 (PMC5862575; doi:10.18632/oncotarget.24284)
Supplement: Supplementary file 1 [file oncotarget-09-13244-s001.pdf]

miR-128 inhibits telomerase activity by targeting TERT mRNA

SUPPLEMENTARY MATERIALS

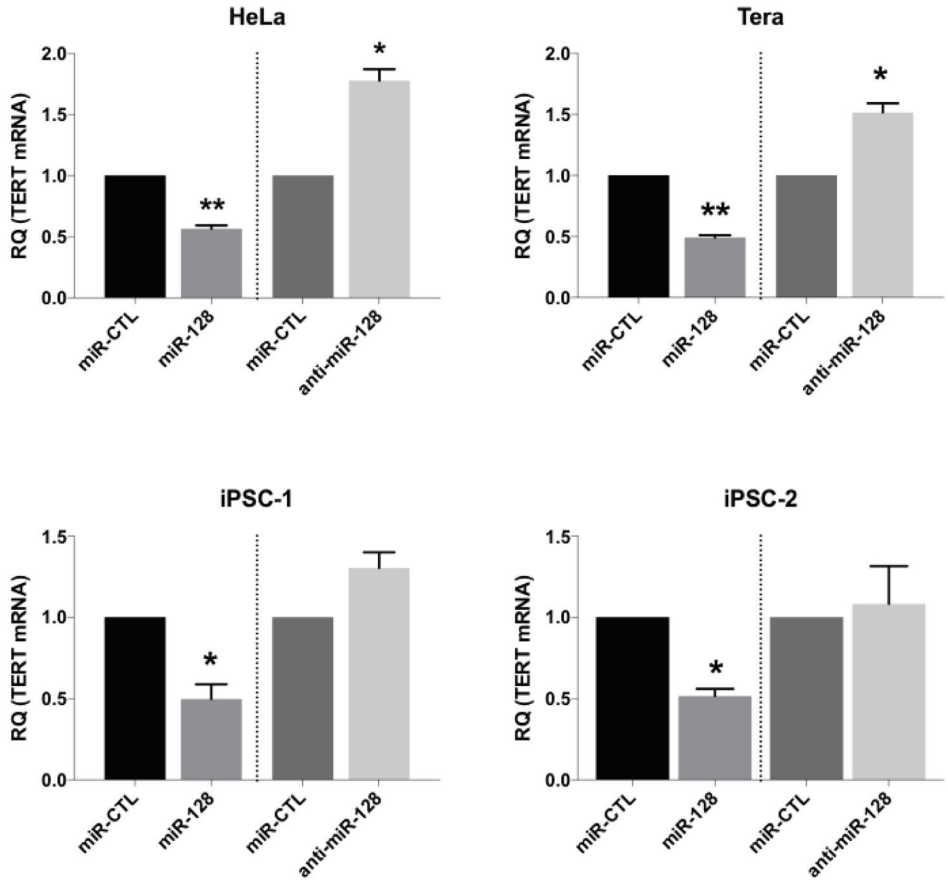

Supplementary Figure 1: miR-128 regulates TERT mRNA levels in HeLa, Teratoma and induced pluripotent stem cells (iPSCs). TERT mRNA expression levels were determined by q-RT-PCR analysis in HeLa cells, in a Teratoma cell line (Tera) and in two different induced pluripotent stem cell lines (iPSC-1 and iPSC-2). Tert levels were normalized to B2M mRNA levels. Results shown as mean ± SEM, n=3, independent biological replicates. $^{*}p < 0.05$, $^{**}p < 0.01$, by two-tailed Student's *t*-test.
